# Bioharvesting and improvement of nano-silica yield from bagasse by irradiated *Curvularia spicifera*

**DOI:** 10.1186/s12866-025-03770-6

**Published:** 2025-02-06

**Authors:** Amira G. Zaki, Samah A. Yousef, Yasmeen A. Hasanien

**Affiliations:** Plant Research Department, Nuclear Research Center, Egyptian Atomic Energy Authority, Cairo, Egypt

**Keywords:** Silica nanoparticles, Solid-state fermentation, Gamma irradiation, Response surface methodology, *Curvularia Spicifera*, Bagasse

## Abstract

**Background:**

Sugarcane bagasse is an organic waste material abundant in silica. Silica is a very significant inorganic substance that is widely employed in a variety of industrial applications.This study displays an eco-friendly and inexpensive biotransformation process producing silica nanoparticles (SNPs) using a primarily reported *Curvularias picifera* strain under solid-state fermentation (SSF) on bagasse as a starting material. The produced SNps were characterized by XRD, DLS, Zeta sizer, FT-IR, SEM, and TEM analyses. The silica bioleaching ability of C. *spicifera *was further amended by exposure to gamma irradiation at a dose of 750 Gy. The biotransformation process was additionally optimized by applying response surface methodology (RSM).

**Result:**

According to screening experiments, the selected promising fungal isolate was identified as *Curvularia spicifera* AUMC 15532. The SNPs fabrication was significantly enhanced by gamma irradiation (optimal dose 750 Gy) and response surface methodology for the first time. The attained SNps’ size ranged from 30.6–130.4 nm depending on the biotransformation conditions employed in the statistical model, which is available for numerous applications. The XRD shows the amorphous nature of the fabricated SNPs, whereas the FTIR analysis revealed the three characteristic bands of SNPs. The outcomes of the response surface optimization demonstrated that the model exhibited an adequate degree of precision, as evidenced by the higher R^2^ value (0.9511) and adjusted R^2^ value (0.8940), which confirmed the model’s close correspondence with the experimental data. A gamma irradiation dose of 750 Gy was optimal for a significant increase in the silica bioleaching activity by C. *spicifera* fermented bagasse (71.4% increase compared to the non-irradiated strain).

## Introduction

The development of procedures enabling efficient NPs production is the focus of scientific interest for numerous research teams [1–4].Fungi-mediated bioprocesses might seem alluring for producing NPs on a broader scale since they are sufficiently resilient to disturbances that may arise during a bioreactor-based process. Due to their filamentous character, they can survive the flow pressure and the mixing parameters [5, 6].

Silica is a significant inorganic substance widely employed in various industrial applications, including fillers, catalytic supports, polymers, molecular sieves, biomedical uses, and resins. Inorganic microstructures with pores are often of interest because they can be used to create mechanically robust encapsulation structures and low-density, thermally stable particles [7].

Although different types of nanoparticles are being used more often in many economic sectors, there is rising concern about the biological and environmental safety of the processes involved in their manufacture. Since, hazardous materials such sodium borohydride, poly-N-vinyl pyrrolidone (PVP), Tetrakis(hydroxymethyl)phosphonium chloride (THPC), and hydroxylamine were utilized in the conventional wet synthesizing methods of nanoparticle. Other dry techniques including aerosol, lithography, and UV irradiation are likewise regarded as environmentally unfriendly [8]. Because hazardous chemicals on the surface of nanoparticles and non-polar solvents limit their applicability in clinical sectors, the usage of such toxic chemicals remains a matter of great concern. Therefore, creating environmentally friendly processes for the synthesis of nanomaterials from bio-based sources including microorganisms, plants, lipids, proteins and various bio-wastes like agricultural residues, vegetable waste, fruit peels, eggshells, and others by employing green nanotechnology and producing the least amount of waste has become a major issue for researchers. Green nanotechnology offers instruments for converting biological systems to environmentally friendly methods of synthesizing nanomaterials. It combines the principles of green chemistry and engineering to synthesize safe and environmentally friendly metal nanoparticles using less expensive materials, consuming less energy, and providing efficient methods for product recycling [9, 10]. Green technology opened the door to creative applications across a range of industries, technologies and processes. These nanoparticles play a leading role in environmental applications like bioremediation, toxin removal, and water purification, as well as agriculture applications like early identification and management of diseases, precision agriculture using nanosensors, increased productivity using nanopesticides and fertilizers, and better food quality and safety via advanced packaging materials, medicinal applications like drug delivery and cancer therapy, biological applications like antimicrobials, textile industry, food industry, electrochemical applications, and others [11, 12].

Silica-based material chemical synthesis is relatively expensive, environmentally dangerous, and frequently needs extremes in temperature, pressure, and pH. On the other hand, under mild physiological conditions, the manufacture of silica occurs by biotas like cyanobacteria, sponges, plants, and diatoms, producing a variety of intricate and hierarchical biogenic nano-silica framework structures [13–15]. The development of alternative biological techniques appears realistic, and in addition to having a significant ecological impact, it would also be financially competitive with currently used technology.

Due to the environmental issues related to agro-industrial wastes, waste recycling has become an attractive topic for academics in chemistry and chemical engineering fields. Silica synthesis from the sugar industry releases ash, which is one of the ways it is recycled [16]. It contains 62.73% silica that can be utilized as a raw material for obtaining mesoporous silica [17]. Solid-state fermentation (SSF) is a complex three-phase process that helps in microbial cultivation for bioprocesses’ enhancement. It involves solid, liquid, and gaseous stages. In the last two decades, SSF has attracted a lot of consideration due to the reduced energy requirements for output products that are more valuable and the need for less waste water discarding with a lower frequency of bacterial contamination. Also, it is an environmentally friendly cultivation method since the substrate is often created from solid waste from agro-industrial systems [18, 19]. Design of experiment (DOE) is a collection of statistically and mathematically planned approaches to obtain more information about the effect of many process variables and their interactions on a defined response (as a product yield) with reduced experimental trials [20–22].

This study aims at developing a green, eco-friendly SNPs production methodology with a considerably low cost through a simple SSF of bagasse (a silica-rich substrate) by an easily manipulated fungal template, *Curvulariaspicifera*. Different gamma irradiation doses were applied to *C. spicifera*to improve the bioleaching proficiency. The conditions of the silica bioleaching process from bagasse, including waste weight, fungal incubation period, reaction pH, and reaction time, were statistically adjusted using response surface methodology for attaining nano-silica at variable particle size range required for different applications, including nuclear application as it could be added to the concrete as a partial replacement of cement to enhance the physical, mechanical and shielding capabilities of concrete in addition, shielding of X-rays and nuclear disposal packaging. Furthermore, silica nanoparticles have been widely employed to absorb uranium ions from nuclear waste.

## Materials and methods

### Isolation of fungal isolates

White sand samples from the Western Desert, Egypt, were collected for fungal isolation. Ten gram-weighted sample was transferred to conical flasks containing 100 ml of sterilized distilled water, and the mixture was left on the shaker at room temperature for 15 min. Different serial dilutions were then prepared, and 100 µl from each dilution was spread on potato dextrose agar (PDA) composed of (g/L): potato infusion 200, dextrose 20, and agar 20. The plates were incubated at 28 ^o^C for the appearance of fungal colonies. The colonies that appeared were picked up and purified on new PDA plates. Pure colonies were then preserved at PDA slants in the refrigerator for the next screening step.

### Screening the fabrication of SNPs by the isolated strains from inorganic source

Primarily, a fungal spore suspension was prepared as follows: cultivated slants were flooded with sterile saline, vigorously vortexed, then serially diluted and counted using a hemocytometer to attain a suspension of 1 × 10^8^ spores/mL.

The purified fungal isolates were screened for nanosilica production from magnesium trisilicate Mg_2_O_8_Si_3_ (an inorganic source of silica). 1 ml spore suspension of each fungal isolate was separately inoculated into flasks containing sterilized potato dextrose broth (PDB) and then incubated for seven days on a shaking incubator (120 rpm at 30 ^o^C). After fungal growth progression, filtration was carried out to separate the fungal biomass from broth media. Accordingly, the harvested fungal biomass was washed twice with distilled water and finally with ethanol and distilled water.

Five grams of wet weight from the collected biomass of each isolate was transferred separately -under aseptic conditions- to a sterilized flask containing 100 ml of 1mM magnesium trisilicate. Flasks were left for three days on a shaking incubator at 100 rpm at 30 ^o^C to allow SNPs synthesis. After the end of the shaking period, fungal biomass was separated through filtration. The resultant filtrate after sonication was examined for SNPs production by DLS analysis.

### Screening the fabrication of SNPs by the isolated strains under solid-state fermentation

Bagasse was obtained from a local shop at Belbes, El-Sharqia, Egypt. Biosynthesis of SNPs from a fermented bagasse is processed here under solid state fermentation (SSF) through the reported method by Zaki et al. [19]. Primarily, SSF was managed as follows: weighted samples of 10 g of bagasse were separately transferred to 250 mL flasks, then a solution of Czapex’s mineral salt composed of NaNO_3_, 0.3; K_2_HPO_4_,0.1; KCl, 0.05; MgSO4・7H_2_O, 0.05; FeSO_4_・7H_2_O, 0.001 (g%, w/v) was added for moistening the waste at a level of 70% w/w then they were autoclaved. After cooling, the flasks were inoculated with 1 mL of the adjusted fungal spore suspension of the selected isolates, followed by a gentle shaking of the flask contents. Flasks were then incubated at 28 °C for eight days.

The next step was for SiO_2_ to leach from the fermented bagasse. Accordingly, 100 mL of sterile distilled water was added to the fermented bagasse, and the biotransformation process of the silica by the tested isolatesinto nano-SiO_2_ was run at room temperature for three days in a 200 rpm shaker. After filtration, the supernatant was sonicated, and the obtained SNPs were examined by DLS analysis.

### Morphological and molecular identification of fungal isolate

The selected fungus was identified by morphological features and molecular characterization in the Moubasher Mycological Centerof Assiut University (AUMC), Assiut, Egypt, using a standard identification manual [23, 24] using PDA media. After an incubation period of 7 days, macroscopic characters (colony diameter, color aspect, and mycelial texture) and microscopic characters (somatic and reproductive microstructures) were observed to identify the fungal isolate.

For molecular identification, the fungal isolate was cultured on Czapek’s agar (CZA) medium and incubated at 28°C for five days [24]. DNA extraction was performed at the Molecular Biology Research Unit, Assiut University, using a Patho-gene-spin DNA/RNA extraction kit (Intron Biotechnology Company, Korea). Polymerase chain reaction (PCR) and sequencing were done with the help of SolGent Company, Daejeon, South Korea. The ITS region of the rRNA gene was amplified using the universal primers ITS1 (forward) and ITS4 (reverse) incorporated in the reaction mixture. Primers have the following composition: ITS1 (5’ - TCCGTAGGTGAA CCTGCGG − 3’) and ITS4 (5’- TCCTCCGCTTATTGATATGC − 3’). The purified PCR product (amplicons) was sequenced with the same primers, incorporating ddNTPs in the reaction mixture [25]. The sequences that were obtained were analyzed using the Basic Local Alignment Search Tool (BLAST) from the National Center of Biotechnology Information (NCBI) website. Analysis of sequences and establishment of phylogenetic trees were done with the help of MegAlign (DNA Star) software version 5.05.

### Investigating the gamma irradiation effect on the silica bioleaching activity

In this experiment, spore suspensions of *Curvularia spicifera *were prepared as described above, distributed in paraffin-sealed vials, and undergone gamma irradiation at doses of 250, 500, 750, and 1000 Gy using ^60^Co Gamma chamber, MC20, Russia, at the Nuclear Research Center (NRC), Egyptian Atomic Energy Authority (EAEA), Cairo, Egypt. The average dose rate was 478.15 Gy h^− 1^ at the time of the experiment. After irradiation, the irradiated suspensions were maintained in dark conditions for 24 h at 7 °C to prevent the occurrence of photo recovery. Then, the irradiated suspension at each irradiation dose was separately cultivated, and the SNPs were fabricated under conditions described in the silica bioleaching section under solid‑state fermentation. Biotransformation flasks were incubated on a rotary shaker (200 rpm), and the samples for further analysis were collected and placed in a freezer (-18 ^o^C).The concentration of silica in such samples was determined by the Heteropoly blue method [26] to define the most effective dose of gamma irradiation that enhanced the SNPs formation. Experiments were done in triplicates.

### Experimental modeling

Statistical modeling for SNPs biosynthesis through a response surface methodology (RSM) using Box Behnken design (BBD) was applied for detecting the optimum levels of the most influential factors that affect the size and stability of fabricated SNPs (based on data obtained from the DLS and Zeta potential analyses) of the fabricated SNPs under SSF by the irradiated *C. spicifera*. The most effective synthesis parameters and their ranges that affect SNPs size were determined by reviewing the literature [27, 28]. The four selected critical parameters were illustrated in (Table 1), and each factor was investigated at different three coded levels (− 1, 0, + 1). For a four-factor design, a total of 27 experimental trials were performed. Each design run was duplicated, and the mean size averages of fabricated SNPs were calculated as the response to the biofabrication process.

**Table 1:** Studied parameters with Box-Behnken design codes and levels

Variables	symbol	Coded and actual values			Unit
		-1	0	+ 1	
**bagasse weight**	A	2	4	6	g
**Fungal incubation period**	B	5	8	11	days
**Reaction pH value**	C	5.0	6.5	8.0	---
**Reaction time**	D	24	48	72	hours

A second-order polynomial model with the coded independent variables (Xi, j) was used to obtain minimized-size SNPs.

(Y) as shown in the following equation below:

$$\:Y = {b_0}\sum\limits_{i = 1}^n {{b_i}{X_i}} + \sum\limits_{i = 1}^n {{b_{ii}}X_i^2} + \sum\limits_{i < l = 1}^n {{b_{ij}}{X_i}{X_j}}$$

Where Y is the response value to be modeled (SNPs mean size), b_0_ is the constant coefficient of the model; X_i_ and X_j_ define the independent variables, b_i_ is the coefficient of linear effect, b_ij_ is the coefficient of interaction effect, b_ii_ is the coefficient of quadratic effect, and n is the number of parameters. An ANOVA (analysis of variance) was conducted to specify the significance of the model. The coefficient of determination R^2^ described how well the polynomial model equation fits the data.

After the practical experiments of BBD were carried out, each flask was filtered with filter paper to separate fungal biomass from filtrate containing fabricated SNPs. Part of the filtrate was sonicated and examined using the DLS analysis to determine the mean particle size. The rest of the resultant filtrate was centrifuged for 20 min at 10,000 rpm, and the collected pellet underwent sequential washing steps by sterile distilled water and centrifugation. Finally, it was left to dry at 65^o^C-adjusted oven for 24 h for further characterization steps.

### Characterization of the biofabricated SNPs

An automated spectrophotometer, the Alpha II Bruker, made in Germany, was used to perform FT-IR measurements in the 4000 –400 cm-1 wavenumber range. X-ray diffraction (XRD) was used to examine the samples’ crystal structures (Shimadzu XRD-6000, Japan). At room temperature, XRD patterns were obtained within a 2θ range of 17° to 90°. With an operating voltage of 50 kV, a current of 40 mA, and a scan rate of 0.8°/min, Cu Kα was the radiation source with a wavelength of λ = 0.15408 nm.

Meanwhile, dynamic light scattering (DLS; Malvern Panalytical, Malvern, UK) was applied to determine the particle size distribution, hydrodynamic radius, and polydispersity index (PDI) of the synthesized samples. Transmission electron microscopy (TEM, JOEL JEM-1400, Tokyo, Japan) at an accelerating voltage of 80 kV and scanning electron microscopy (SEM, JEOL JSM-5600 LV, Tokyo, Japan) and were also used to obtain data on the size and shape of the particles.

### Statistical analysis

A free trial version of Minitab software was used to construct and analyze the statistical design of BBD. While, for analyzing the effect of gamma irradiation on the SNPs yield (results were expressed as mean ± standard deviation), SPSS software version 22 (IBM Corp, Armonk, New York, United States) was conducted. Since, the one-way ANOVA and Duncan’s test for comparing means at the 0.05 significance level are used to determine the statistical significance.

## Results

### Screening the SNPs biofabrication by the isolated fungi

**Fig. 1:**
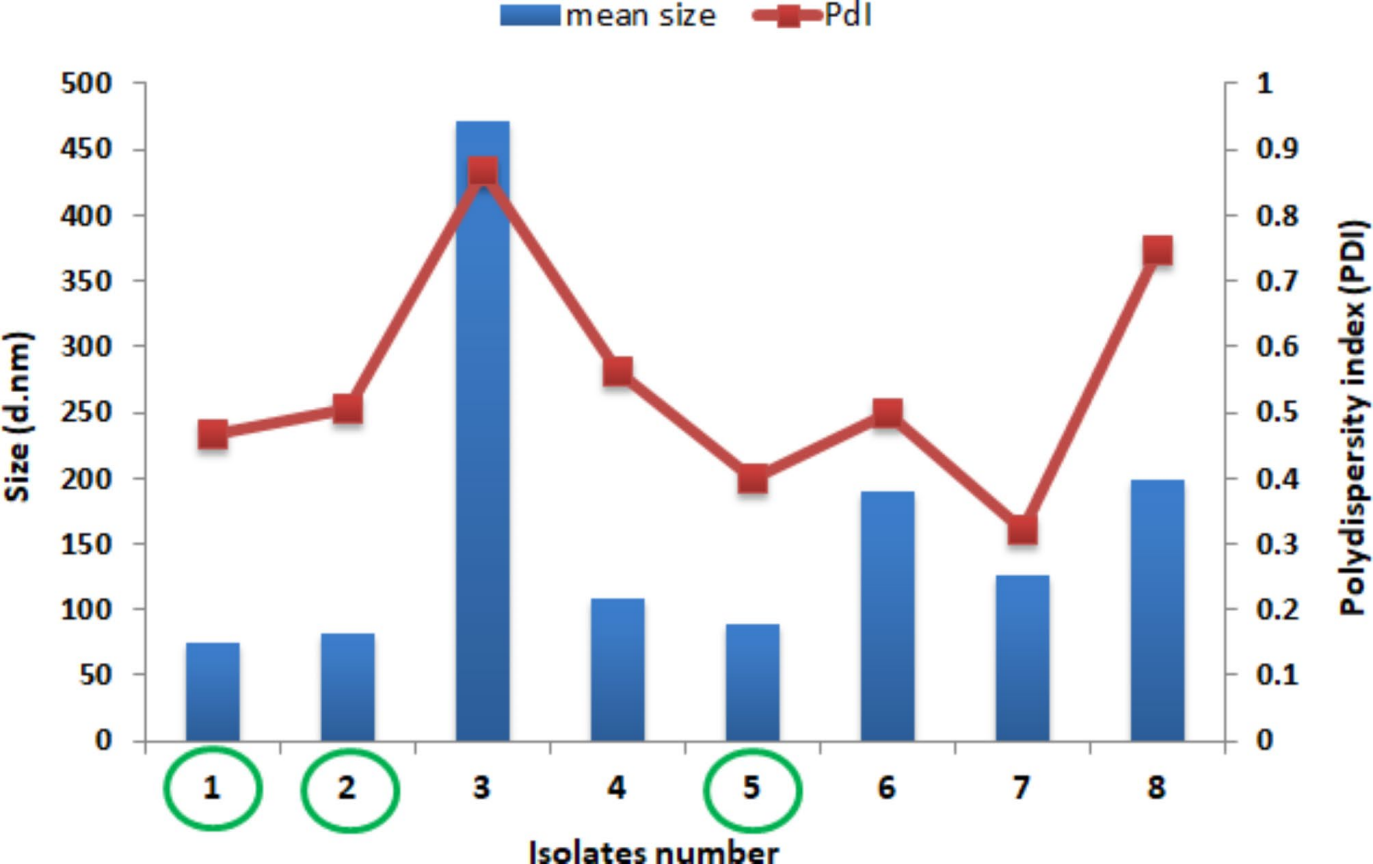
Mean size and polydispersity index of fabricated SNPs by the tested isolates using Mg_2_O_8_Si_3_ as inorganic source of silica

Eight isolates from the isolated fungi produced SNPs within the average nanosize using Mg_2_O_8_Si_3_ as an inorganic silica source. DLS analysis was used to evaluate particle size distribution and to calculate the polydispersity index (PDI) of the biofabricated SNPs, as shown in Fig. 1. It is clear from the results that the fungal isolates number 1, 2, and 5 have the lowest size particles (74.08, 82.44 and 88.78 nm, respectively) and also possess PDI ≤ 0.5.

**Fig. 2:**
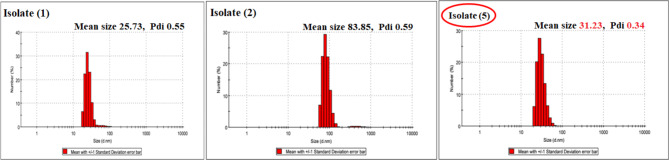
DLS analysis for the biofabricated SNPs by the screened three isolates using bagasse as organic silica source

Therefore, these three isolates were selected for screening for their ability to produce SNPs using bagasse as an organic source of silica under SSF fermentation. Isolate number 5 gave SNPs with an average size 31.23 nm, with the lowest PDI (0.34), indicating the monodispersity of SNPs as shown in Fig. 2. Therefore, this isolate was selected for further experiments after its complete identification (morphological and molecular).

### Morphological and molecular identification of the selected isolate

**Fig. 3:**
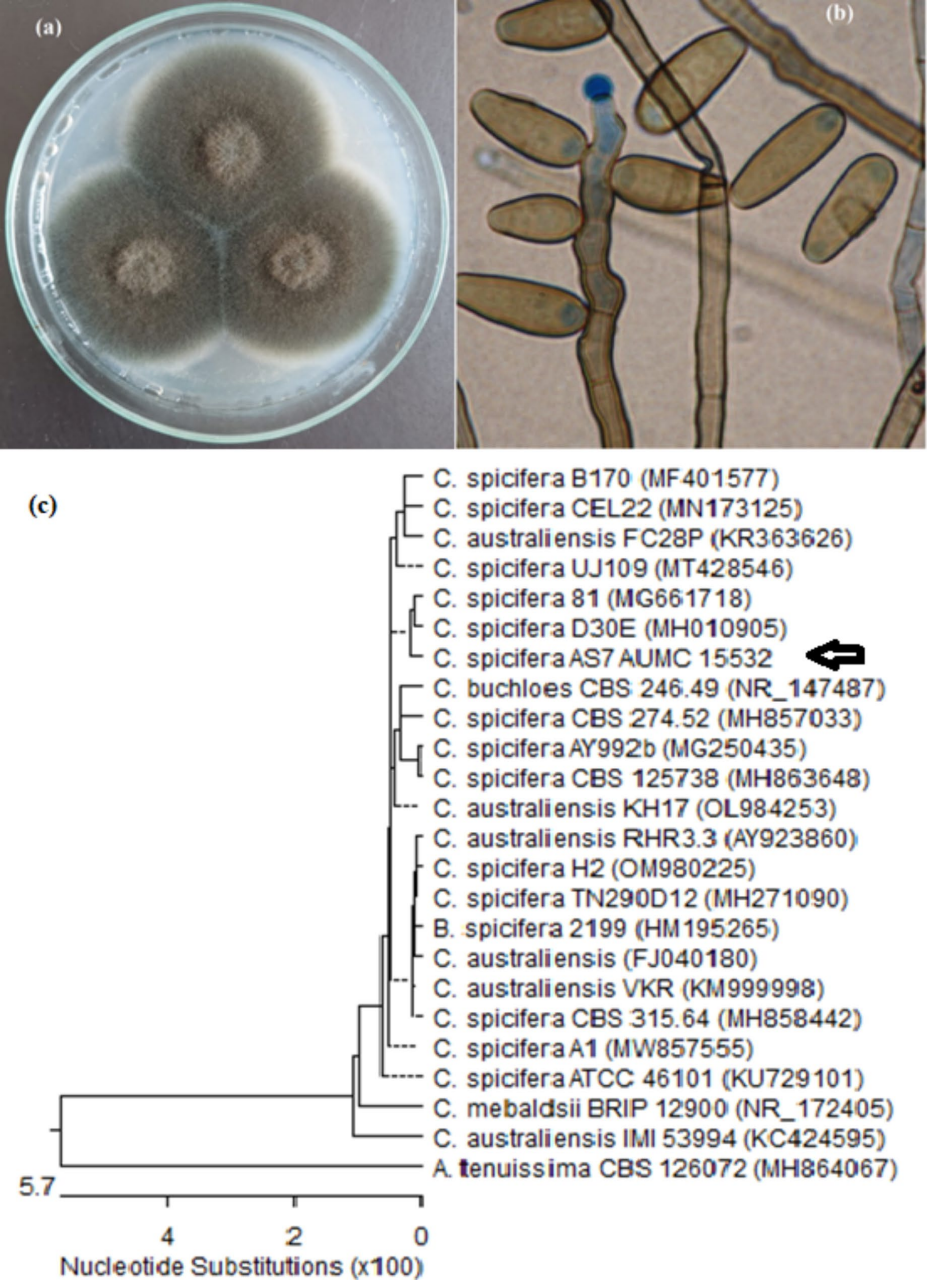
**(a, b)** Macroscopic and microscopic features of AUMC15532 on PDA, **(c)** is the phylogenetic tree based on ITS sequences of rDNA of the fungal sample isolated in the present study (*Curvularia spicifera* AUMC15532, arrowed) aligned with closely related strains accessed from the GenBank. It showed 99.82 -100% identity to *Curvularia spicifera*

The selected promising fungal isolate was identified in AUMC and deposited in their culture collection as *Curvularia spicifera *AUMC 15532.The colony morphology and the microscopic examination under a trinocular light microscope with a camera supply (NOVEL, model: XSZ-107T) were illustrated in Fig. 3 (a and b).Colonies are fast growing, grey to blackish-brown with a black reverse, suede-like to downy in surface texture. Conidiophores are brown, erect, straight to flexuous, septate, and smooth-walled, up to 150 μm long, mostly 3–7 μm wide.

Phylogenetic tree based on ITS sequences of rDNA of the fungal sample isolated in the present study (*Curvularia spicifera* AUMC15532, arrowed in Fig. 3c) aligned with closely related strains accessed from the GenBank. It showed 99.82 -100% identity coverage with several strains of *C. spicifera.*

### Effect of gamma-irradiated *C. Spicifera* on the biofabrication of SNPs

**Fig. 4:**
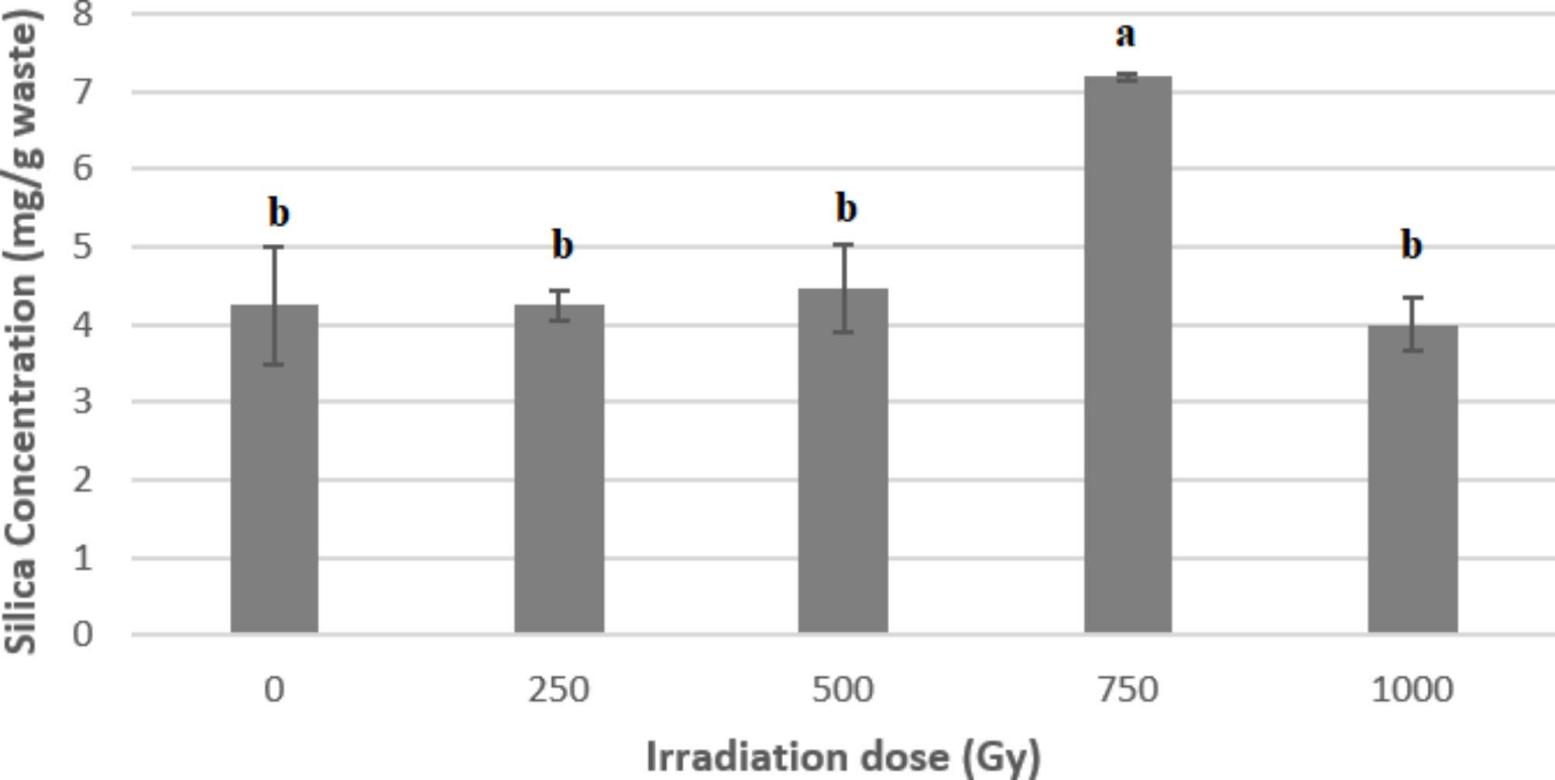
Influence of the different doses of gamma irradiation on the concentration of SNPs produced by *Curvularia spicifera*under SSF with bagass as organic silica source

The tested fungal cells were exposed to different gamma rays by doses 0, 250, 500, 750, and 1000 Gy (each irradiation dose was tested separately), and the obtained results are represented in Fig. 4. According to the findings, 750 Gy was the best irradiation dose that significantly increasedthe biofabrication activity of SNPs. Since, at this dose, the calculated SiO_2_ amount (7.2 ± 0.57 mg/g bagasse waste) was higher significantly (*P* < 0.05) compared to that attained by the non-irradiated culture (4.2 ± 0.57 mg/ g bagasse waste). Silica concentration was calculated using a calibration curve prepared according to the recommended procedure.

### Box-behnken design

In this investigation, the authors used an efficient RSM modeling approach based on a three-level BBD with three variables to reveal the influence of the chosen parameterson the size of the SNPs. Optimization is highly desired to see the effect of the various SSF conditions on the size and stability of fabricated SNPs.The conditions applied for optimizing SNPs biofabrication by the irradiated *C. spicifera* (at 750 Gy) along with experimental (actual) and predicted SNPs size were recorded in Table 2.

**Table 2:** Box- Behnken design matrix showing zeta potential besides actual and predicted DLS-size measurements for silica NPs synthesized via *Curvularia spicifera* at different designed runs

Run no.	Factors				Zeta potential (- mv)	Mean size of SNPs (nm)		
	A	B	C	D		actual	predicted	
1	2	5	6.5	48	24	50.7	54.77	
2	6	5	6.5	48	12.3	100.5	106.38	
3	2	11	6.5	48	14	71.25	63.46	
4	6	11	6.5	48	0.0215	84.37	78.39	
5	4	8	5	24	23.3	74.21	76.85	
6	4	8	8	24	18.5	80.06	78.70	
7	4	8	5	72	19.5	75.24	74.69	
8	4	8	8	72	21	92.26	87.71	
9	2	8	6.5	24	19.5	80.67	82.24	
10	6	8	6.5	24	6.53	95.83	93.93	
11	2	8	6.5	72	13.8	54.7	64.10	
12	6	8	6.5	72	20.8	113.0	118.93	
13	4	5	5	48	6.67	54.86	52.30	
14	4	11	5	48	18	68.8	71.13	
15	4	5	8	48	15.8	83.06	88.23	
16	4	11	8	48	7.93	40.03	50.09	
17	2	8	5	48	10.2	30.64	28.87	
18	6	8	5	48	7.96	93.29	93.17	
19	2	8	8	48	13.1	72.85	67.35	
20	6	8	8	48	13.4	73.42	69.57	
21	4	5	6.5	24	13.3	72.84	68.86	
22	4	11	6.5	24	19.5	105.7	108.70	
23	4	5	6.5	72	0.036	130.4	121.78	
24	4	11	6.5	72	19.4	64.28	62.64	
25	4	8	6.5	48	16.8	86.38	87.69	
26	4	8	6.5	48	16.5	89.00	87.69	
27	4	8	6.5	48	16.65	87.69	87.69	

A: bagasse weight, B: incubation period, C: pH, D: reaction time

**Fig. 5:**
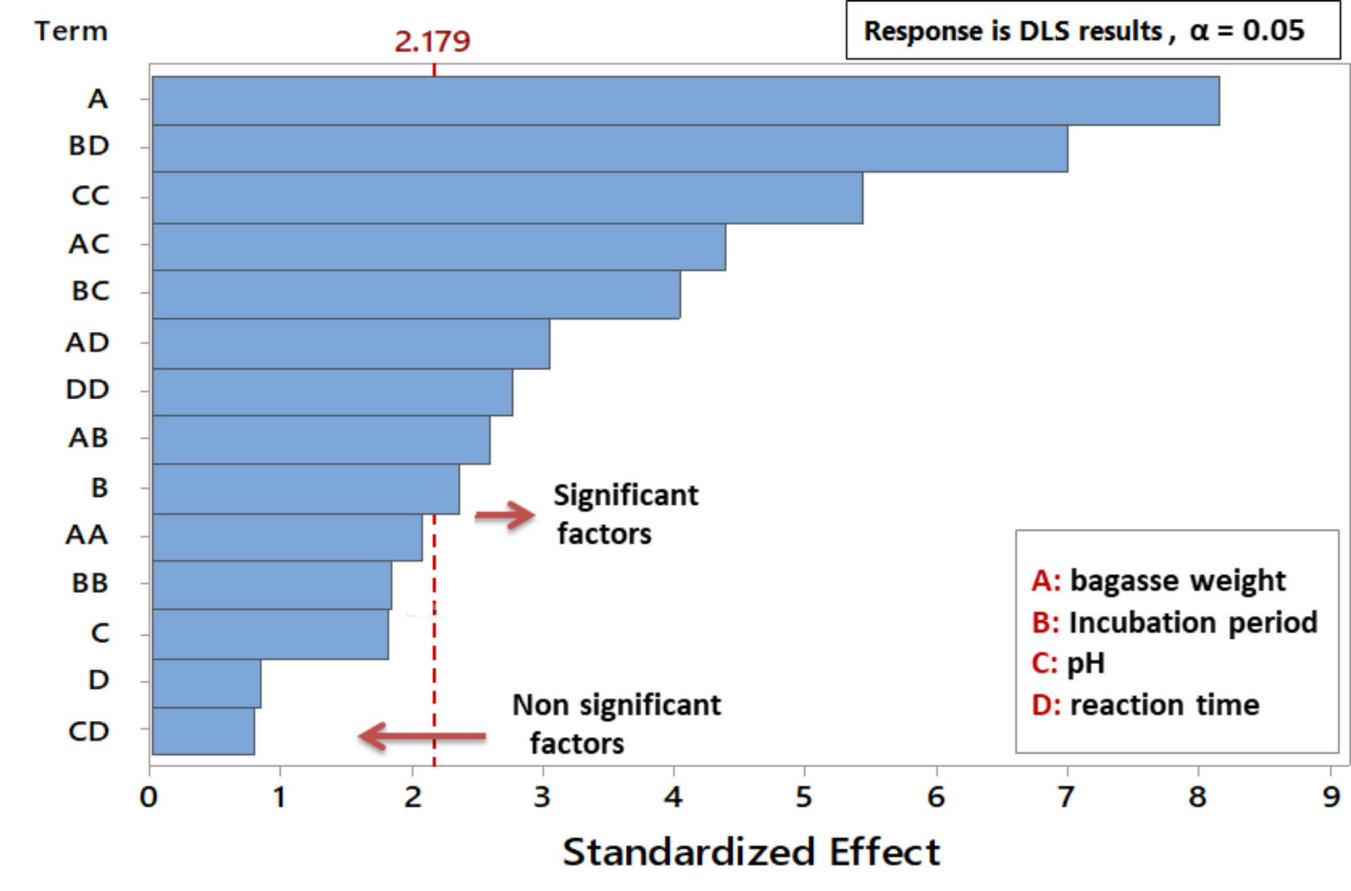
Pareto chart of the standardized effects on the mean size of SNPs fabricated by *Curvularia spicifera *under SSF with bagasse as organic silica source, showing signifcant and non-signifcant factors

The model’s significance and suitability were evaluated using analysis of variance (ANOVA) with Fisher’s statistical analysis; the outcomes are displayed in Table 3. The model was significant as the model *P* value was less than 0.05. Additionally, the model terms A, B, BD, AB, AD, AC, BC, D^2^, and C^2^ were shown to be significant based also on values obtained for the *P* value (< 0.05), and they were visually represented in a pareto chart in Fig. 5. An increased R^2^ value of 0.9511 and an adjusted R^2^ value of 0.8940 confirm that the model has a high degree of precision and that the suggested model closely matched the experimental data. The polynomial equation represented the relationship between the response and the factor under study and was produced by the model as follows:

**Table 3:** Analysis of variance (ANOVA) for the estimated response (mean size of fabricated silica nanoparticles)

Source	DF	Adj SS	Adj MS	T-Value	F-Value	*P*-Value
Model	14	11669.8	833.56	-	16.67	0.000
Linear	4	3801.0	950.25	-	19.00	0.000
A	1	3320.0	3320.01	8.15	66.38	0.000
B	1	279.7	279.66	-2.36	5.59	0.036
C	1	166.1	166.06	1.82	3.32	0.093
D	1	35.3	35.26	0.84	0.70	0.418
Square	4	2811.9	702.97	-	14.05	0.000
A^2^	1	212.7	212.75	-2.06	4.25	0.062
B^2^	1	168.4	168.43	-1.84	3.37	0.091
C^2^	1	1474.7	1474.67	-5.43	29.48	0.000
D^2^	1	379.1	379.05	2.75	7.58	0.018
2-Way Interaction	6	5056.9	842.82	-	16.85	0.000
AB	1	336.4	336.36	-2.59	6.72	0.024
AC	1	963.5	963.48	-4.39	19.26	0.001
AD	1	465.3	465.26	3.05	9.30	0.010
BC	1	811.4	811.40	-4.03	16.22	0.002
BD	1	2449.3	2449.26	-7.00	48.97	0.000
CD	1	31.2	31.19	0.79	0.62	0.445
Error	12	600.2	50.02	-		
Lack-of-Fit	10	596.8	59.68	-	34.78	0.028
Pure Error	2	3.4	1.72	-		
Total	26	12270.0		-		
	**R**^**2**^ **= 95.11%**,** R**^**2**^**(adj) = 89.40%**					

A: bagasse weight, B: incubation period, C: pH, D: reaction time,

The analysis of variance (ANOVA) was applied at 95% confidence intervals; variablesand models would be statisticallyconsiderable at levels of significance, **P** value < 0.05. (-) T value indicates negative effect, (+) Tvalue indicates positive effect, DF: degree of freedom, adj SS: adjusted sum of squares, adjMS: adjusted mean of squares, F Value: ratio of two variance, P Value: probability value, R^2^: determination coefficient, R^2^(adj): adjusted determination coefficient

$$\eqalign{\:{\text{MeanSize}}\:\left( {{\text{nm}}} \right) & = - 709 + 56.0\,{\text{A}} + 51.56\, {\text{B}} + 140.8\,{\text{C}} \cr &+ 0.013\,{\text{D}}  - 1.579\,{{\text{A}}^2} - 0.624\,{{\text{B}}^2} - 7.39\,{C^2} \cr & + 0.01464\,{{\text{D}}^2}  - 1.528\,{\text{AB}} - 5.17\,{\text{AC}} + 0.2247\,{\text{AD}} \cr & - 3.165\,{\text{BC}}  - 0.3437\,{\text{BD}} + \:0.0776\,{\text{CD}}  \cr}$$

**Fig. 6:**
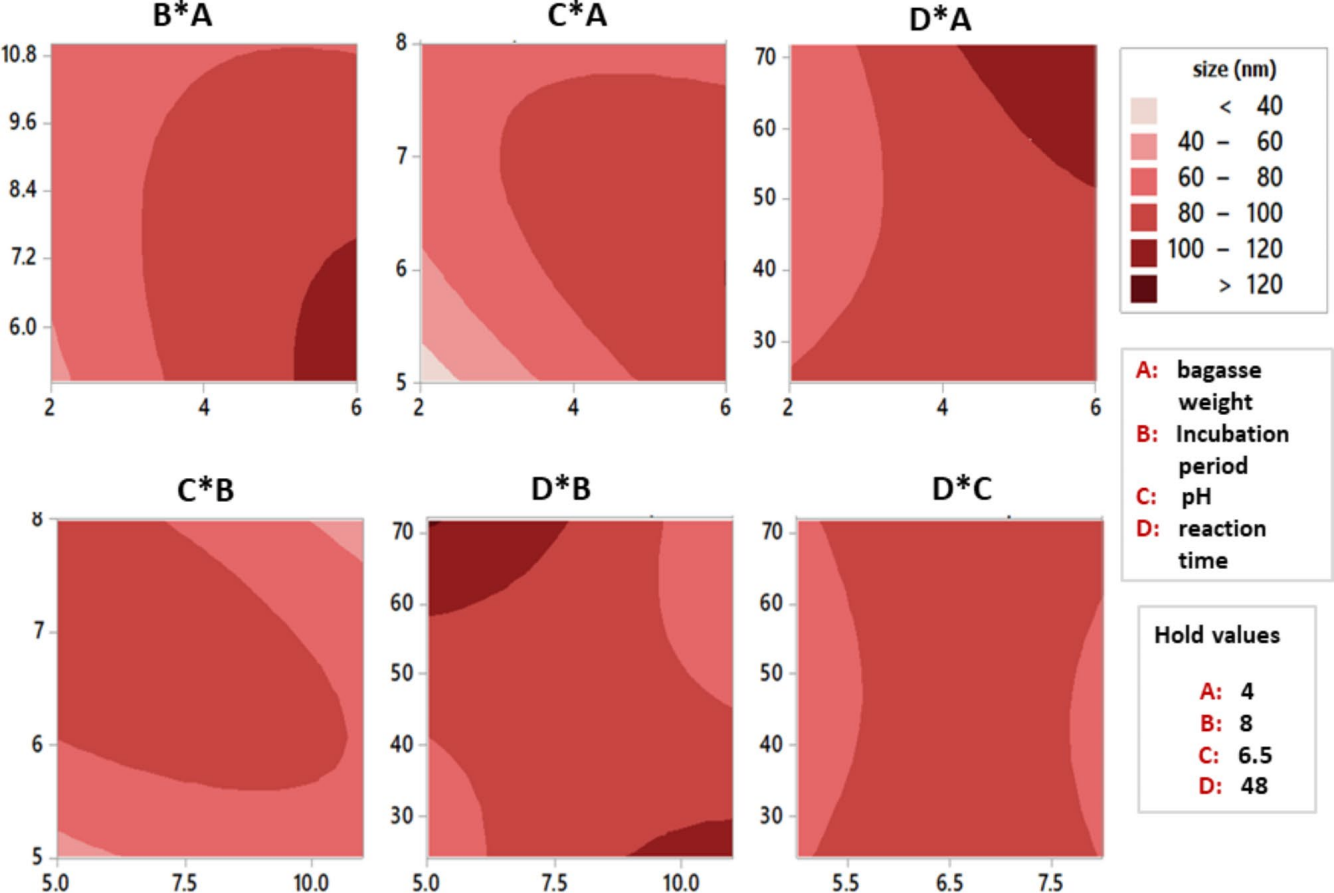
contour plots (2D) show the interaction effect of different factors on size of SNPs fabricated by *Curvularia spicifera* under SSF with bagasse as organic silica source

The contour plot visualizes the relationship between predictor and regressed variables. It displays a two-dimensional image in which all spots with comparable responses are linked together to form contour lines with constant responses. It consists of three components: (a) predictors on the x and y axes, (b) a contour line that connects points with the same response, and (c) contour bands of the same color that represent ranges of the response variable. The contour plot in Fig. 6 reveals the link between tested parameters that influence the SNP biofabrication process. The lighter zone represents smaller SNP sizes, which is desirable. Generally, the plots demonstrate that the interaction between associated variables was significant and helpful for optimizing the biofabrication process of SNPs. From this model analysis, the process conditions of run 17 (Table 2) are optimum values for obtaining SNPs at the desired small size of 30.64 nm. Moreover, this model provide an adjusted plateform for controlling and predicting different required size ranges of the fabricated SNPs.

### Characterization of the biofabricated SNPs

**Fig. 7:**
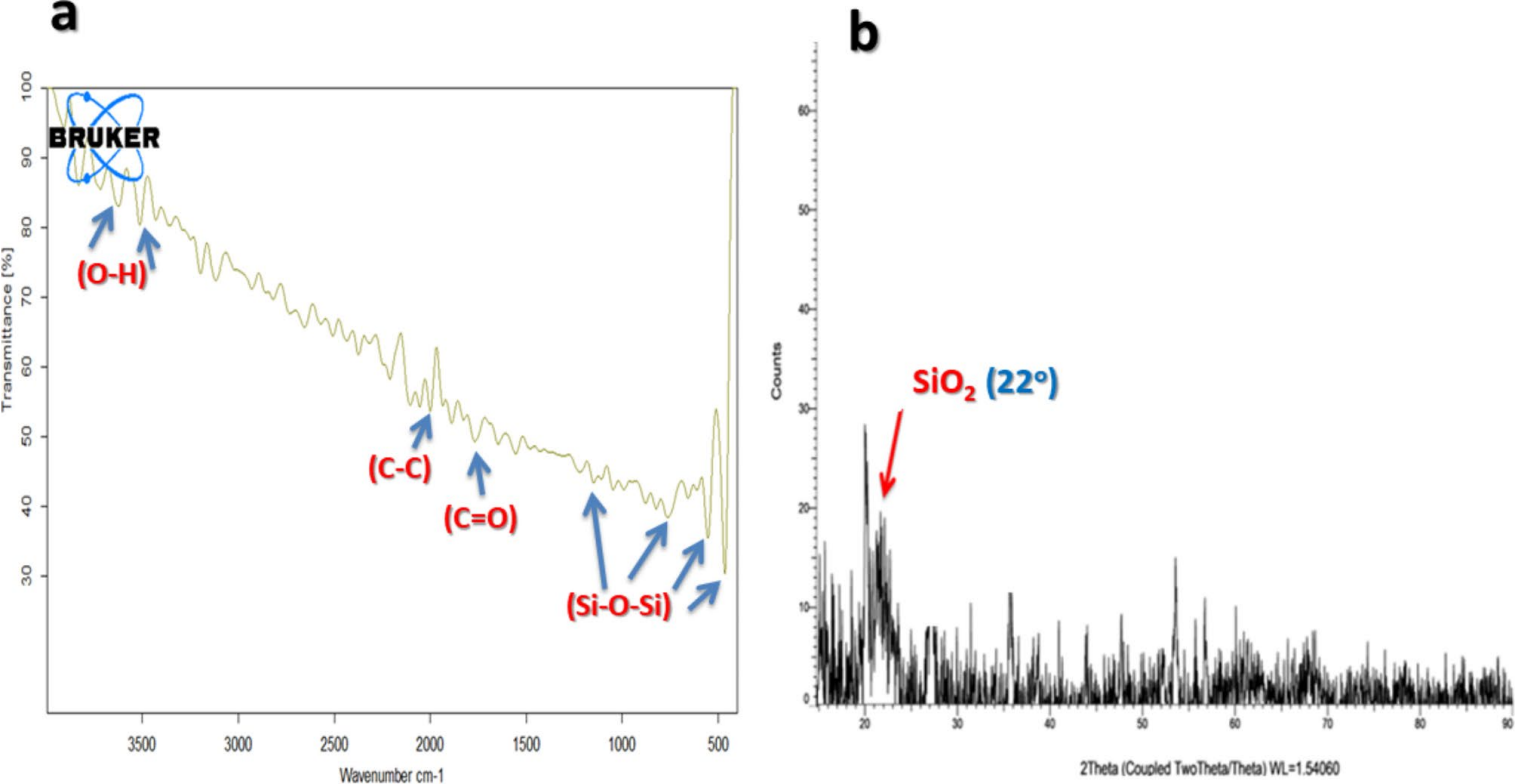
Characterization of the biofabricated SNPs under SSF by *Curvularia spicifera* **(a)** FT-IR spectrum, **(b)** XRD spectrum

The dried SNPs obtained of SSF of Bagasse by *Curvularia spicifera* were tested by FTIR, XRD, SEM, and TEM analysis. FTIR spectrum (Fig. 7a) shows bands at 2035 cm^− 1^ and 1715 cm^− 1,^ which is attributed to organic components (organic carbon and fatty acids, respectively) that form silica nanoparticles.The presence of OH group, organic carbon, and water molecules, respectively. The band at 3500 and 3680 cm^− 1^ is caused by the (OH) stretching vibration of the water adsorbed on the surface of the SNPs. The peak at 478 cm − 1 is due to the Si–O bending mode of vibration, while silica is shown a band at 1070 cm^− 1^ associated with asymmetric stretching vibration of siloxane bond (Si–O–Si). Amorphous silica exhibits a relative peak of around 800–600 cm^− 1^.

**Fig. 8:**
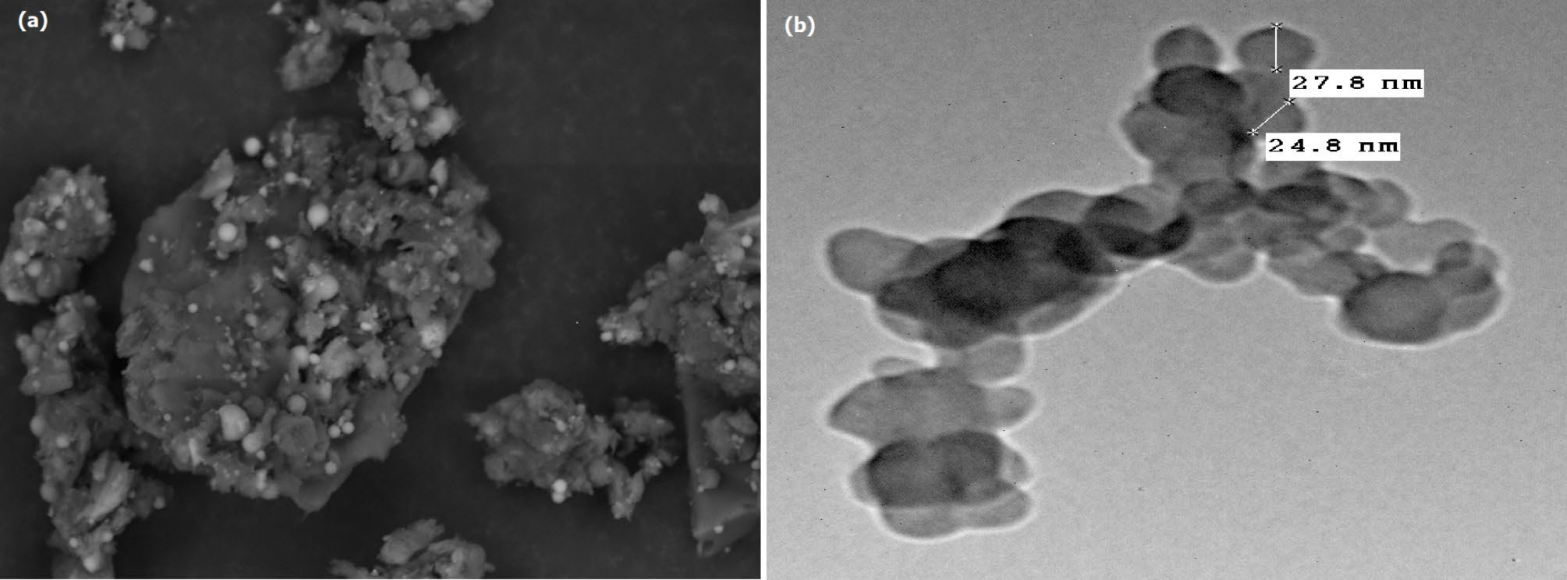
Electron microscope imaging of the biofabricated SNPs under SSF by *Curvularia spicifera ***(a)** Scanning electron microscope, and **(b)** Transmission electron microscope

XRD patterns of the synthesized SNPs are illustrated in Fig. 7b. The spectrum revealed a broad scattering maximum centered at 22°, corresponding to amorphous silica. The electron microscopic image of the biosynthesized SNPs is exhibited in Fig. 8. As seen in Fig. 7a, SEM images of the biofabricated SNPs reveal particle spheres with a smooth surface and semi-homogeneous size. Each spherical particle has a distinct grain nanosize and is mixed in with the fungal medium.The produced SNPs had a spherical structure with diameter sizes ranging from 24.8 nm to 27.8 nm, according to the TEM image shown in Fig. 8b.

## Discussion

As the population grows, more land is available for agriculture, which raises the proportion of organic waste in agriculture. Organic wastes are often disposed of by being dumped in landfills, which increases pollution and has detrimental effects on the ecosystem [29, 30]. Waste buildup contaminates natural resources, including water, soil, and air, and leads to several environmental issues. In addition, it raises the risk of epidemics and medical pandemics and endangers public health. As a result, scientists emphasize developing the most effective technology to recycle organic wastes and use them as raw materials in other industries’ production chains [15].

Most studies are interested in the production of nanocellulose from bagasse, but there are few about the production of nano-silica, so the interest in this study was on the production of nano-silica to expand the benefit from recycling sugarcane waste. Since, sugarcane is one of the primary potential sources of silica extraction and subsequent transformation on nanostructured silicon among the various natural resources available. This feedstock is utilized to produce ethanol and sugar, with bagasse—a byproduct of the process—serving as the primary energy source. According to estimates, 250 kg of bagasse (containing 50% humidity) and 6 kg of ash (equal to 2.5%) are produced for every ton of sugarcane [31]. Also, the applied solid state fermentation technique in this study may provide a valuable platform for the creation of novel nanostructures with a wide range of applications through recycling of various plant wastes.

Sugarcane bagasse is an agricultural organic waste used to generate energy in industrial ovens worldwide, but its value has skyrocketed due to its relevance as a recyclable material. It can potentially be used as a natural source of silica extraction and then turned into nanostructured silicon [15, 32]. Egypt is responsible for producing more than 16.8 million tons of sugarcane residue annually [33]. The recycling of sugarcane bagasse is a promising sector in Egypt since it offers a significant possibility for the creation of environmentally friendly building materials and products that are used daily.

Sugarcane bagasse, a cellulose fiber left behind after harvesting the sugar-bearing juice from the sugarcane plant, is a significant supply of silica (containing approximately 50–97% w/w) for silica nanoparticle production [34]. Silica nanoparticles have significant industrial, biotechnological, and biomedical/pharmaceutical uses because of their ability to be easily shaped, small size, compatibility with living organisms, large surface area, and ability to be modified for various purposes. These characteristics have resulted in widespread use in different fields [35]. Due to their nontoxicity and biocompatibility, they play a significant role in biological applications such as cancer imaging, bone tissue engineering, drug delivery, and biosensors [36–38].The progress of scientific research has brought about a significant transformation in all aspects of our lives, including healthcare and agriculture.

The current study’s objective is to enhance the SNPs biofabrication by *C. spicifera *from bagasse with minimal expense by using SSF, statistical optimization, and gamma radiation. Related investigations used agricultural wastes high in silica for silica biosynthesis [39, 40]. Additionally, several microorganisms have been used to biologically synthesize Si/SiO_2_nanocomposites from rice husks and wheat bran at room temperature [41].

**Fig. 9:**
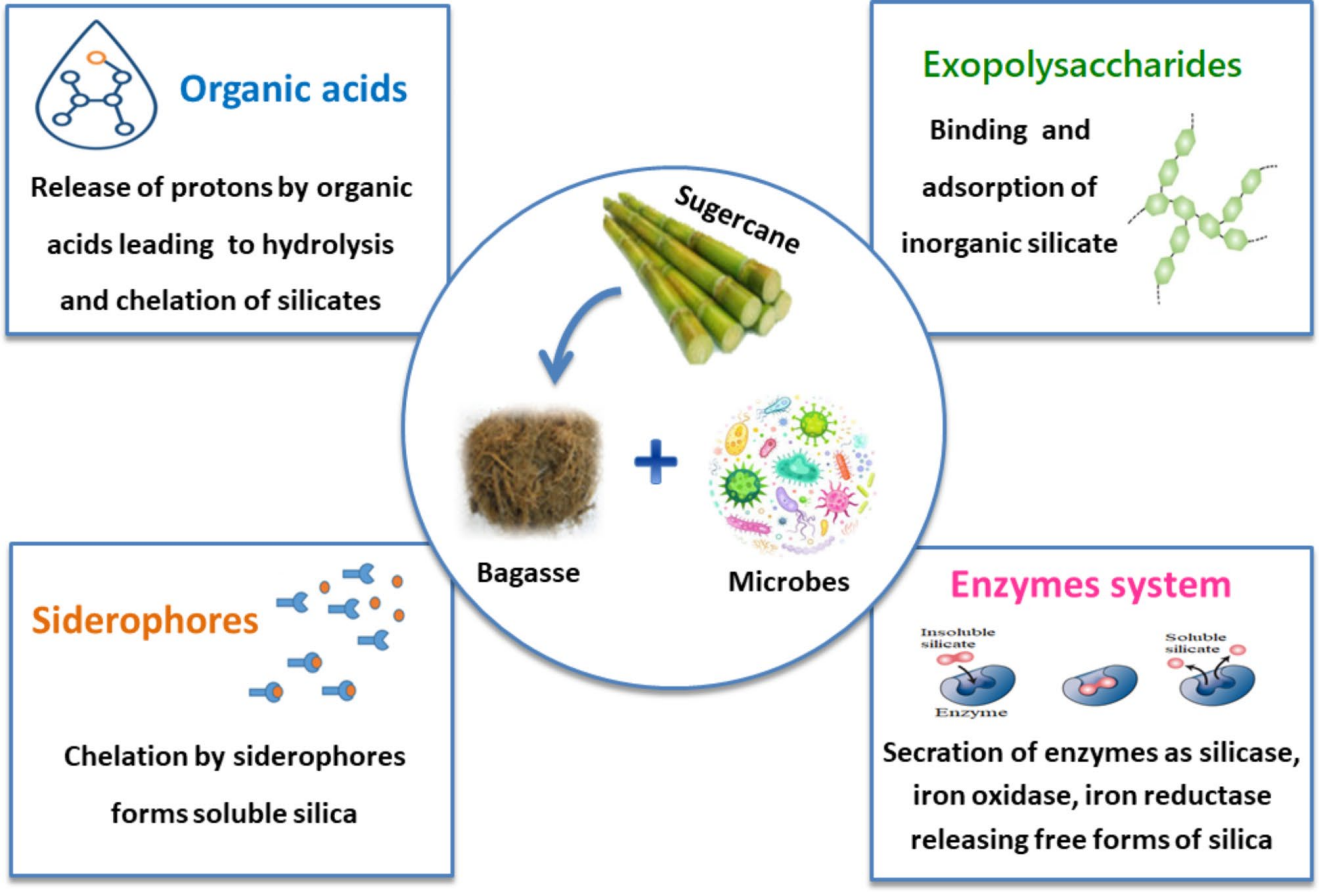
Predicted mechanisms for microbial fabrication of silica nanoparticles

Expected mechanisms for microbial synthesis of silica nanoparticles are presented in Fig. 9. It is believed that generally, the fabrication of silica nanoparticles happens in two stages as a result of the bioconversion process of agricultural waste using microbial bioactivity. Primarily, the dissolution of insoluble silicate from waste releases silicic acid through the secreted organic acid. Secondly, silicic acid is hydrolyzed by specific extracellular microbial enzymes into silica, which is then released as nanoparticles into the reaction medium [42–44].

Microbes hydrate respiratory CO_2_ to make organic acids, such as acetic, citric, oxalic, and gluconic acids. These acids bind to iron and aluminum in waste materials containing silicates, releasing the silicates in soluble form [45].Organic acids provide protons (H^+^) for two purposes: protonation, which facilitates silicate hydrolysis, and chelation, which promotes silicate dissolution through complexation with cationic components of silicates.

Certain microorganisms can produce several substances, including exopolysaccharides and siderophores, which can enhance the process of bioleaching silica from its sources. Organic acids have a great affinity for polysaccharides, resulting in forming an area that contains a high concentration of organic acids close to minerals. The presence of organic acids led to the partial dissolution of minerals, while polysaccharides could absorb SiO_2_ [46]. Siderophores are low molecular weight chelators that strongly attract divalent and trivalent metals. Siderophores are believed to play a role in the solubilization of silicates in acidic environments, as silicate materials contain various elements, such as iron and aluminum. The proposal suggests that siderophores bind to metal ions, causing the release of Si from silicates [47, 48].

SSF is preferable to other methods due to its many advantages, including decreased catabolite repression and substrate inhibition, more effective enzyme harvests and volumetric outcomes, low energy consumption, prolonged product stability, no release of organic wastewater, and low production costs. Additionally, using agro-industrial wastes as substrates makes this method more environmentally and economically beneficial with a greater potential for application in pharmaceutical and biomedical industries [49]. Fungal cells under solid state fermentation could produce organic acids such as citric acid and lactic acid as well as enzymes such as amylases; xylanase; cellulases; proteases; pectinases; phytases; laccase; lipases; ligninases, and secondary metabolites such as gibberilic acid, alkaloids [50, 51].

Piela and colleagues suggested an enzyme-based system for bioleaching silica nanoparticles by fungi. When stressed fungal cells come into touch with a substrate, they release specific proteins and enzymes that react with the biomass’s silica structure to form an enzyme-silicic acid combination. The hydrolytic enzymes released by fungal cells further break down this complex, releasing siliceous groups found in the substrate as silica nanoparticles [52]. Khan and coworkers previously suggested a similar process [53].

Numerous microbial cells can be used to synthesize nanoparticles. Each one can be used to effectively produce certain metallic or metallic oxide nanoparticles, with varied degrees of biological processing capabilities. Because of the activity of their enzymes and innate metabolic processes, not all microbial cells are able to produce nanoparticles. Therefore, to create nanoparticles with precise characteristics like size and morphology, careful selection of the right microbial cell is required. In literature, metallic nanoparticle synthesis is most likely to occur in microbial cells that have the capacity to accumulate heavy metals. Additionally, methods for cultivating are also necessary. Therefore, it is possible to greatly boost enzyme activity by optimizing culture conditions like medium, buffer strength, inoculum size, incubation period, mixing speed, temperature, light, and other conditions [54]. Here, the criteria used to select fungal cells for the biotransformation procedure depending primarily on the ability of the fungal cell to synthesize silica nano particles (SNPs) from the inorganic Mg2O8Si3 source as well as possessing low polydispersity index (PDI) ≤ 0.5. Accordingly, three fungal strains (Strain 1, strain 2, strain 3) were primarily selected for their ability to synthesize nanosilica with different nanosize of 74.08, 82.44 and 88.78 nm, respectively and also possess PDI ≤ 0.5. The subsequent criteria that was taken into consideration was the nanosilica leaching ability of the three selected fungal strain from bagasse as an organic source under SSF fermentation. Accordingly, isolate number 5 identified as *Curvularia spicifera* gave SNPs with an average size 31.23 nm, with the lowest PDI (0.34). Additionally, different biotransformation process conditions were also optimized.

The effectiveness of green synthesis of nanoparticles is still mainly limited to the lab stage. Large amounts of homogenous nanoparticles cannot currently be produced by laboratory-controlled biosynthesis since batch-to-batch variations can occur. Scalable, continuous flow-based synthesis are thus desperately needed. Although there has been progress in manipulating the morphologies of nanoparticles, there is still a lack of control over their dimensions, especially size via the green synthesis methos. The benefits of nanoparticles sizes are restricted to a specific range. Also, It can be challenging to effectively utilize biological agents for nanoparticles’ synthesis due to the inadequate understanding of the underlying mechanics of many biosynthesis processes, particularly microbial synthesis. Because of this, synthesis techniques are inconsistent and unpredictable [55].

Literature concerning the application of biocatalytic strategy to synthesize nanosilica particles and these procedures are based almost exclusively on the enzymatic activity of *Fusarium* strains [56–58]. There is little information on using microorganisms to synthesize nanosilica from bagasse. Thus, the current research is focused on evaluating a viable biocatalytic approach that results in the synthesis of silica nanoparticles from bagasse. Pineda and colleagues [59] investigated the biocatalytic activity of *F. oxysporum* on rice husk ash (RHs ash), which was employed as a biotransformation substrate. As a result, after the fifth day of the reaction, the fluid test sample (2 g of RHs ash, 0.5 g of wet biomass, 100 mL of distilled water) had approximately 2.7 times more silica than the control samples (2 g of RHs ash, 100 mL of distilled water). Zielonka and coworkers [60] demonstrate that the test samples (consisting of 4 g of RHs, 10 g of *Aspergillus parasiticus*, and 100 mL of distilled water) achieved five times more silica compared to the control samples (consisting of 4 g of RHs and 100 mL of distilled water).

To evaluate responses following the adjustment of numerous process parameters, statistical optimization techniques such as response surface methodology (RSM) are frequently utilized [20, 61]. Here, the nanosilica produced by *C. spicifera* showed a more significant response to variations in the examined parameters like pH, reaction time, incubation period, and bagasse weight using the response surface methodology. It successfully identified the ideal concentrations needed to fabricate nanosilica with various sizes and excellent stability. The response surface plots offered a straight forward and practical means of demonstrating the primary and secondary effects of the critical parameters on the utilized biofabrication process. They also defined the ideal concentrations for efficient synthesis of SiO_2_ NPs at the necessary nanosizes under SSF, with the highest stability indicated by high zeta potential values.

Box-Behnken’s design (BBD) can identify nonlinearity and interactions between the studied process variables. It is a flexible model and provides fewer experimental runs, which helps to save a significant amount of time, energy, and resources. It is frequently used to optimize the response surface of experiments, which do not require a full three-level factorial experiment and instead use a second-order (quadratic) model for the response variables. BBD is regarded as an improved version of incomplete block designs in two-level factorial design. Additionally, BBD takes into account the region of interest (design space) and the region of operability (explorable space) on the same plane, which offers a number of benefits for design space prediction. Other beneficial aspects of the BBD include its excellent design qualities, which include minimal collinearity and orthogonality [62].

According to the International Standards Organizations (ISOs), it has been determined that PDI values below 0.05 are more commonly associated with monodisperse samples. In contrast, values beyond 0.7 are typically associated with a broad size distribution of particles, alhgo known as poly-disperse [63]. There was a moderate amount of mono-size dispersion among the produced particles in the nanocomposite, as indicated by theobtained PDI values. As previously stated, quasi-spherical crystalline silica measuring 2–6 nm and 2–8 nm were obtained by Bansaland Pineda-Vasquez [56, 58], respectively. In contrast, the experiments described in this article enabled the gathering of silica NPs comprising a broad range of nanosizes, depending on the reaction conditions. These results are consistent with the data obtained by Zielonka [60].

In literature, silica nanoparticles were leached from rice husk using *Fusarium oxysporum* in a submerged state. This method produced highly crystalline particles of 2–6 nm quasi-spherical capped by stabilizing proteins [56]. Another study synthesized nanosilica using a fermented rice husk by *Fusarium oxysporum* under solid state fermentation. XRD analysis showed a wide scattering peak centered at 2θ = 22.7° reflecting the amorphous nature of the particles. TEM image and DLS analysis revealed the spherical shape of the particles and polydispersion of the particles with a mean size 69.4 nm [19]. Another related studies reported the green synthesis of nano-silica using *Rhus coriaria L.* extract and sodium metasilicate (Na_2_SiO_3_.5H_2_O). Their synthesized nanoparticles were spherical, polydisperse, and somewhat agglomerated with a size range of 55 nm. XRD analysis showed a broad peak centered at 2θ = 23° with relatively low noise, reflecting their amorphous nature. In addition, the synthesized particles recorded a zeta potential of -45 mV, reflecting the particles’ stability [64]. Another study used lemon peel extract and Na_2_SiO_3_ for nano silica synthesis. The XRD spectra of their synthesized particles had peaks that correspond to the planes 2θ = 28.4°and also indicated the crystalline nature of the particles with a calculated diameters of 42.6 nm. Also, TEM image showed the circular shape of particles [65]. Another study used an aqueous extract of *Punica granatum* leaves and tetra ethyl ortho silicate as a precursor for nanosilica synthesis. The synthesized particles were spherical in shape and amorphous in nature with an average size of 12 nm [66].

Gamma radiation is a short wave of high-energy electromagnetic radiation that might cause specific mutations. Exposing of microbial cells to ionizing radiation initiated a chain of processes that resulted in physiological alterations and antibacterial activity. These adjustments are depending on the absorbed dose. Thus, radiation causes additional stress in the cells, which disrupts their structure [67]. Here, a gamma irradiation dose of 750 Gy was optimal for the significant increase in the silica bioleaching activity by *C. spicifera-*fermented bagasse (69.41% increase compared to non-irradiated strain).

Gamma rays are suggested in a number of studies in the literature as a physical mutagen to enhance microbial cultures and boost their nanoparticles’ production capacities [19, 67]. Notably, the use of gamma irradiation mutagenesis to improve microbial cultures may result in the separation of hyper-producers, which would reduce the process’s overall cost [68]. When microbial cells were exposed to ionizing radiation, a sequence of events occurred that resulted in alterations in physiology and antimicrobial activity. The absorbed dose serves as the basis for these modifications. Radiation causes the cells to undergo more stress, which tends to disrupt their arrangement. Irradiated cells exhibit a variety of alterations as a result of this metabolic disruption, some of which are temporary and some of which are permanent. It has been demonstrated that low irradiation dose and dose rate can promote the induction of antioxidant defense mechanisms, including catalase (CAT), superoxide dismutase (SOD), glutathione (GSH), and glutathione peroxidase (GSH-Px) [67].

In conclusion, the benefits of solid-state fermentation (SSF), rarely documented in nanoparticle synthesis, were exploited in this investigation. The low moisture level of the culture used in SSF encourages the production of extracellular enzymes and active chemicals by the microorganism, which are crucial to synthesizing NPs. Furthermore, SSF utilizes the entire culture (biomass and fungal metabolites) in NP synthesis to produce an effective biosynthesis process. This SSF-dependent technique is environmentally friendly and cost-effective because it commonly uses solid waste from agro-industrial systems as a substrate. Furthermore, this study discovered a previously unknown biological approach for producing nano-SiO_2_ from bagasse using *C. spicifera*. Furthermore, gamma-irradiated *C. spicifera* at a dose of 750 Gy demonstrated higher bioactivity than the non-irradiated strain. As a future work, preparation of nanosilica thin film from the currently synthesized nanosilica in this study will be processed and subsequent studying its potentiality in the field of food industry to wrap foods for protecting them from the external conditions, and microbial infection.

## Data Availability

Data availability statement: the datasets analysed during the current study are available in the NCBI GenBank data base repository with the accession number of PP837581, https://www.ncbi.nlm.nih.gov/nuccore/PP837581.
